# A pilot clinical trial on a Variable Automated Speed and Sensing Treadmill (VASST) for hemiparetic gait rehabilitation in stroke patients

**DOI:** 10.3389/fnins.2015.00231

**Published:** 2015-07-10

**Authors:** Karen S. G. Chua, Johnny Chee, Chin J. Wong, Pang H. Lim, Wei S. Lim, Chuan M. Hoo, Wai S. Ong, Mira L. Shen, Wei S. Yu

**Affiliations:** ^1^Department of Rehabilitation Medicine, Tan Tock Seng Hospital Rehabilitation CentreSingapore, Singapore; ^2^Division of Electronic and Computer Engineering, Biomedical Engineering Centre, Ngee Ann PolytechnicSingapore; ^3^Clinical Research Unit, Tan Tock Seng HospitalSingapore, Singapore

**Keywords:** stroke, treadmill, hemiplegia, gait, sensing, automated

## Abstract

**Introduction:** Impairments in walking speed and capacity are common problems after stroke which may benefit from treadmill training. However, standard treadmills, are unable to adapt to the slower walking speeds of stroke survivors and are unable to automate training progression. This study tests a Variable Automated Speed and Sensing Treadmill (VASST) using a standard clinical protocol. VASST is a semi-automated treadmill with multiple sensors and micro controllers, including wireless control to reposition a fall-prevention harness, variable pre-programmed exercise parameters and laser beam foot sensors positioned on the belt to detect subject's foot positions.

**Materials and Methods:** An open-label study with assessor blinding was conducted in 10 community-dwelling chronic hemiplegic patients who could ambulate at least 0.1 m/s. Interventions included physiotherapist-supervised training on VASST for 60 min three times per week for 4 weeks (total 12 h). Outcome measures of gait speed, quantity, balance, and adverse events were assessed at baseline, 2, 4, and 8 weeks.

**Results:** Ten subjects (8 males, mean age 55.5 years, 2.1 years post stroke) completed VASST training. Mean 10-m walk test speed was 0.69 m/s (*SD* = 0.29) and mean 6-min walk test distance was 178.3 m (84.0). After 4 weeks of training, 70% had significant positive gains in gait speed (0.06 m/s, *SD* = 0.08 m/s, *P* = 0.037); and 90% improved in walking distance. (54.3 m, *SD* = 30.9 m, *P* = 0.005). There were no adverse events.

**Discussion and Conclusion:** This preliminary study demonstrates the initial feasibility and short-term efficacy of VASST for walking speed and distance for people with chronic post-stroke hemiplegia.

## Introduction

Despite advances in acute stroke care and a plethora of rehabilitation therapies, 45% of stroke survivors have residual impairments following stroke, 90% have some form of functional disability and 50% are unable to regain premorbid walking abilities and capacity. The loss of independent walking ability after a stroke is a predictor for discharge to nursing homes (Gresham et al., 1975; Holden et al., 1986; Portelli et al., 2005).

Locomotor deficits observed following stroke have a profoundly negative impact on functional independence, mobility, community participation and quality of life. Gait and mobility impairments correlate with higher fall risks, osteoporosis and higher energy costs (Eng and Tang, 2007). Walking also has important implications for health in the older adult population as walking ability may also provide protective effects against secondary late post stroke complications such as osteoporosis, falling, and heart disease (Eng and Tang, 2007). Slow walking speed, inability to ambulate 1 mile (1609 m) and inability to walk one flight of stairs contribute to greater frailty and disability in older adults (Gill et al., 2006). Hence, for those undergoing rehabilitation, regaining walking abilities feature prominently in patient's stated functional goals (Bohannon et al., 1988).

Regarding retraining of motor and walking function, exercise strategies may be subdivided into neurodevelopmental or neurofacilitation techniques, strength training, task-specific treadmill training with or without body weight support and intensive mobility training. For the physiotherapist involved in treadmill training, this could be considered as “forced use” therapy, which maximizes the load bearing and activation of paretic leg muscles through intensive, repetitive stepping, improved reflexive gait patterning using altered afferent proprioceptive input and augmented locomotor entrainment with task repetition. Such inputs are crucial for motor learning and progression of metabolic demands through varying velocity, training duration, and programme length for stroke survivors (Ivey et al., 2008). Treadmill training (with or without body weight support) is at least as effective as other forms of gait training (Moseley et al., 2005; Eng and Tang, 2007). Treadmills with bodyweight support may allow lower functioning individuals to undertake early intensive walking practice in a less effortful manner.

For treadmill training or mechanically assisted walking training without body weight support, a recent meta-analysis by Polese (Polese et al., 2013) showed that, for subjects with subacute and chronic stroke, treadmill training resulted in a faster, and larger walking distance, when compared to no intervention/non-walking intervention, These effects were maintained for as long as 12 weeks following the intervention period. No significant difference was found between treadmill training and overground training for distanced walked. Other advantages of treadmill training include promotion of hip extension, reduction of compensatory gait patterns and discouragement of hip circumduction.

The use of standard treadmills to train hemiplegic subjects poses treatment challenges due to several factors such as the lack of automated safe speed progression and backward displacement with fixed belt speeds which may increase fall risk. To mitigate this risk, 1 or 2 therapists are often needed to ensure patient safety during training and to facilitate hip swing in weaker hemplegic subjects. When a safety harness is not employed concurrently with treadmill training, the physiotherapist may sometimes assume awkward postures, putting them at risk of repetitive strains or unanticipated injuries.

Standard treadmills also suffer from a lack of relevant feedback to the hemiplegic patient with regard to foot placements on the moving belt or the use of excessive arm supports. Other limitations which may negatively affect desired training intensities using standard treadmills include the fact that lowest standard speed settings are generally challenging for moderately gait -impaired stroke subjects who may have difficulty in keeping up with a pre-set treadmill speed when fatigue sets in during training, thus increasing fall risk.

This study was conducted to test a novel Variable Speed and Sensing Treadmill (VASST) treadmill prototype jointly developed by bioengineers and clinicians using a standard clinical protocol. Preliminary feasibility, acceptability and safety were the main primary outcomes of this study. The primary hypothesis was that a 4 week period of VASST training could result in a 20% gain on either self-selected gait speed or walking distance.

## Materials and methods

### Study design

A feasibility clinical study of 10 subjects with chronic stroke was conducted in an ambulatory rehabilitation facility with direct links to a tertiary rehabilitation center and acute hospital with a dedicated stroke unit. The duration of the study period was from 1 April 2012 to 30 June 2013. Ethical approval from institutional review boards was obtained prior to subject recruitment. All subjects signed informed consent prior to research interventions. All subjects were recruited consecutively from the outpatient rehabilitation clinics and the majority of subjects had completed their inpatient stroke rehabilitation programme at the center's rehabilitation unit (NCT number 01811680, NHG DSRB D 2012/00571).

### Subjects

Subjects were included if they fulfilled the following inclusion criteria: first ever clinical stroke (ischemic or hemorrhagic) confirmed on CT or MR brain imaging, aged between 21 and 80 years, duration of >3 months post stroke, ability to walk over ground at a self-selected speed of 0.1 m/s or faster with or without walking aids or orthoses, for at least 150 m with contact guard or supervision (Functional ambulation category 2 or more) (Holden et al., 1986).

Exclusion criteria included: (1) medical conditions limiting fitness for treadmill training such as uncontrolled hypertension/hypotension, Diabetes Mellitus, angina pectoris, recent myocardial infarction, congestive cardiac failure, known echocardiographic ejection fraction < 40% within 3 months of recruitment, chronic arrhythmias (e.g., atrial fibrillation) within 3 months of study recruitment and presence of a cardicac pacemaker; (2) End stage illness (advanced malignancy), pregnancy or end stage renal failure with life expectancy of < 6 months; aphasia or disorders of communication (inability to obey 2 step commands), severe cognitive impairment, (Folstein Mini Mental State Examination <21/30, dementia, untreated depression or psychiatric disorder) (Folstein et al., 1975), (3) active lower limb arthritis, presence of lower limb pain (Visual Analog Scale) >5/10, fixed orthopedic deformities of the lower limb which would compromise safe ambulation on treadmill; (4) moderate to severe lower limb spasticity or spasms (Modified Ashworth Scale > 2); and (5) conditions which could be exacerbated by pressure effects of the gait safety harness such as active skin lesions over the trunk, abdominal aortic aneurysm and hypercoagulable states or anticoagulation.

### Engineering aspects of VASST

**Figure 1:**
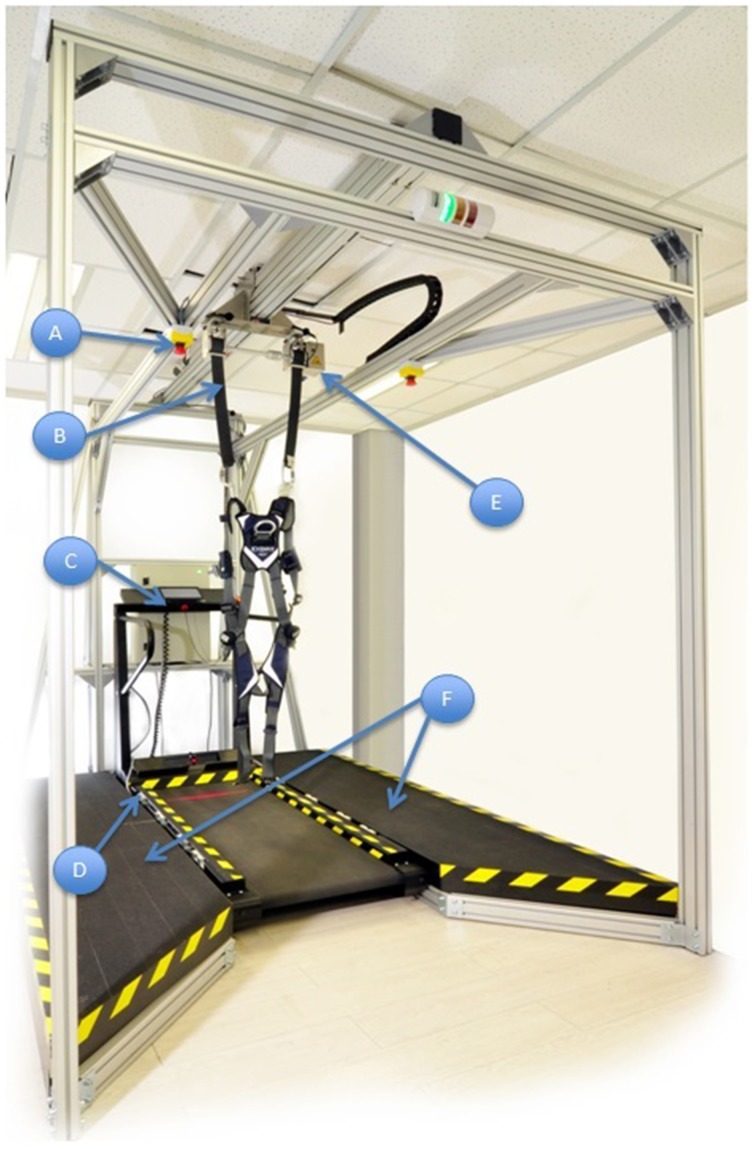
**The variable automated speed sensing treadmill is a semi-automated integrated rehabilitation system with multiple sensors and microcontrollers**. (a) Additional emergency stop buttons. (b) A fall prevention harness with sensors for detection of fall motion, with automatic relay to the main controller to stop the treadmill. (c) Pre-programmed exercise parameters within Micro controller system, which can be adjusted to optimize training of the subject. (d) Laser beam foot sensors to detect subject's walking gait on the belt and modify the exercise speed according to a preset algorithm. (e) Wireless control to reposition the harness to be overhead of the subject's position on the belt. (f) Flanking platforms for the physiotherapist to stand beside either side of the subject.

The Variable Automated Speed and Sensing Treadmill is a semi-automated integrated rehabilitation system with multiple sensors and microcontrollers. Its key features include:

- A fall prevention harness with sensors for detection of fall motion, with automatic relay to the main controller to stop the treadmill.
- Wireless control to reposition the harness to be above the subject's position on the belt.
- Flanking platforms allowing standing room on either side of the subject.
- Additional emergency stop buttons.
- Pre-programmed exercise parameters, which can be adjusted to optimize training of the subject.
- Laser beam foot sensors to detect subject's walking gait on the belt and thus modify the exercise speed according to a preset algorithm.
- Software tools displayed on a panel at the head of the treadmill to collect data on ambulatory gait speed, distance and time walked (Figure 1).

**Figure 2:**
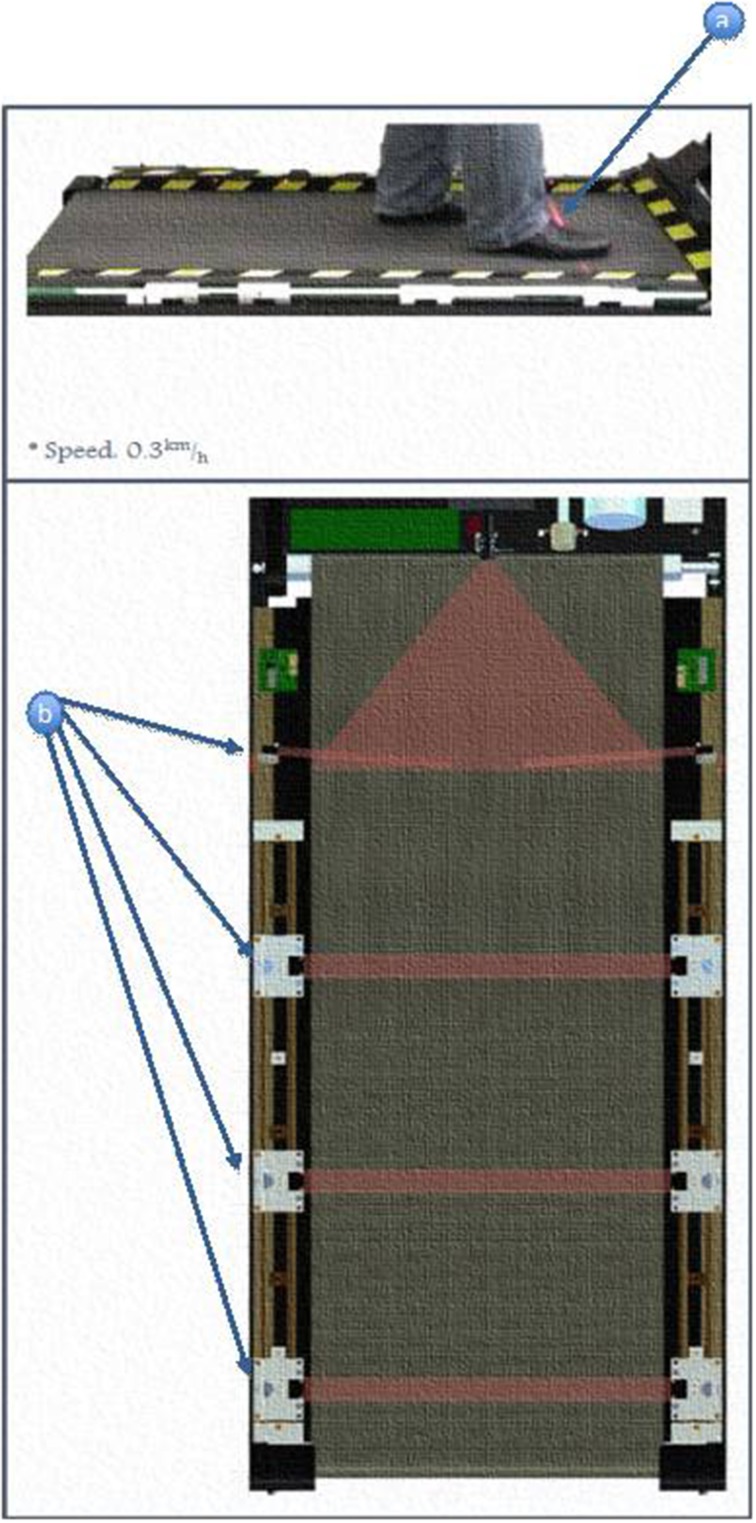
**Positioning an activation of laser beam sensors**. (a) Subjects's foot triggers the motion of the treadmill. (b) Different locations of the light beam sensor.

**Figure 3:**
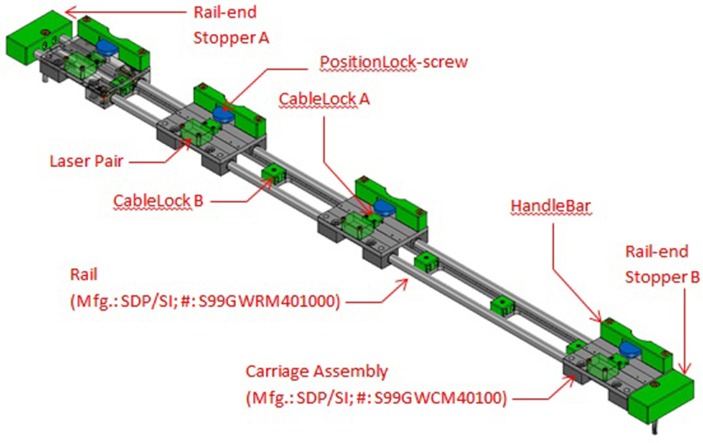
**Details of sensor carriage assembly showing the four carriages for the four sensor beams**. The carriages may be repositioned to different locations and locked in place.

As a first step, a standard rehabilitation treadmill, GaitKeeper GK2000T (from Mobility Research), was modified and enhanced in the following manner:

- A 32-bit (ARM4) microcontroller was used to replace the manufacturer's controller. Our microcontroller interfaced directly with the treadmill's motor driver and took direct control over the belt movement. It stored the exercise parameters and performance measurements during exercise, and executed the exercise program to train patients. At the end of the exercise, data could be uploaded via Bluetooth to a personal computer. It executed the training algorithm, according to the parameters set by the physiotherapist for each patient.
- Four cross-beam laser sensors were placed across the treadmill to sense foot movements. Interruptions of laser beams resulted from patient behavior in the following manner (Figure 2):
  Beam A was triggered when the patient was able to cope with the exercise speed. It was also used as a trigger to start/restart the training.
  Beam B was triggered when the patient step length was sufficiently wide enough when walking.
  Beam C was triggered when the patient failed to match the belt speed.
  Beam D, if triggered, will cause the treadmill to stop immediately.
  By using a carriage assembly with adjustable positions that could be locked along the length of the assembly, the beam locations could be adjusted according to the subject's stride length (Figure 3). A detailed description of the engineering aspects of VASST is available (Chee et al., 2014).

### Research interventions

A standard VASST training protocol was employed for all 10 subjects and this consisted of a total of 12 sessions of treadmill training on VASST consecutively over 4 weeks (three times/per week). Each session lasted for between 50 and 60 min inclusive of set-up warm-up, training walks and rest breaks within each session. While various studies have described varying frequencies of treadmill training ranging from 10 to 48 sessions over 24 months, we opted for 12 sessions to assess acceptability, feasibility, and safety (Moseley et al., 2005; Eng and Tang, 2007; Ivey et al., 2008). Close supervision was provided by a senior physiotherapist and bioengineer for subject training and VASST operations, respectively. Blood pressure and baseline heart rate were measured prior to and upon completion of each treadmill exercise session. Exercise was halted if subjects felt unwell, experienced chest pains, excessive exertional dyspnoea, or severe limb pain at any time during VASST training.

Prior to application of the safety harness, gentle lower limb stretching exercises were performed. After donning the safety harness, subjects commenced VASST training warm up over a 5 min duration in which they were incrementally brought up to their initial treadmill training speed. The initial VASST speed was calculated at 50% of their baseline 10-m walk tests (10 mWT) walking speeds and thereafter, 50% of their last concluded treadmill training speed for subsequent sets of treadmill walks. A total of three training epochs of 10 min each was conducted with 3–5 min rest breaks in between each epoch. Subjects were allowed to hold either one of the treadmill handlebars at the start of each training session, until they were confident of their balance before releasing their hand-holds.

A speed-dependent treadmill training protocol was employed in the following manner. VASST subjects were instructed to keep walking toward the front of the treadmill system in order to trigger the two proximal foot sensor positions, represented by beams A and B (Figure 2). If these foot placements were detected 60–70% of the time over 1 min, the VASST speed was automatically increased by 0.1 km/h for the next 1 min. If the subject could not keep up with the preset VASST training speed and the distal undesired two foot sensors, beams C and D were triggered 60–70% of the time during the 1 min, the treadmill speed was kept at the same speed for the next minute. If the subject was still unable to keep up during this minute, the speed was automatically adjusted downwards by 0.1 km/h for the next 1 min. If subjects continued to regress backwards and rear-most foot sensors, beam D was triggered, VASST would halt to avoid a potential fall. Such progressions were repeated until the training epoch was completed. Subjects were allowed to rest for 3–5 min prior to commencement of the next training epoch.

### Outcome assessments

All outcomes were measured at four time points including baseline (week 0), midway of training (week 2), completion of training (week 4), and at 1 month follow up (week 8) and they were performed by independent physiotherapists.

Primary outcome measures included measures of self-selected gait speed using the timed 10-m walk test (10 mWT) (m/s) and walking capacity (distance) using the 6-min walk test (6 mWT). All tests were done without physically aiding the subjects but with consistent walking aids or orthoses as required by subjects. Scores were calculated based on the average of two trials. The 6 mWT distance walked was measured using a single timed distance on self-selected indoor level ground (loop of 21.5 m) (Enright, 2003).

Secondary outcomes included measures of overall balance ability using the Berg Balance Scale (BBS) and the Functional Ambulation Category (FAC). BBS is a widely used clinical test consisting of 14 items, graded from 0 (unable) to 4 (independent) with a total score of 56 which can be easily administered over 20 min to test static and dynamic balance abilities in elderly or frail individuals (Blum and Korner-Bitensky, 2008). The FAC (range 0–5) is a functional walking test that evaluates ambulation ability applicable to stroke patients. This 6-point scale assesses ambulation status by brief observation over a distance of 10 feet and determination of the amount of human support the patient requires when walking, regardless of whether or not a personal assistive device is used (Holden et al., 1984).

Gait specific measures of hemparetic step length (HSL) and non-hemiparetic step length of the unaffected lower extremity (NHSL) were recorded at week 0 and week 4 (post training) using the Gaitrite walk way. The Gaitrite is an established portable walkway system used to objectively measure temporal-spatial measures of gait.

Lastly, user acceptability for both patients and physiotherapists was assessed using a self-rated questionnaire administered at the end of training (week 4) in the following manner. For research subjects, the following questions were asked. (1) In the past 4 weeks, did you benefit from training on VASST? Responses were rated as yes/no/neutral. (2) Would you like to have more sessions on VASST in future during physiotherapy sessions? Responses were rated as yes/no/neutral. (3) Please rate your VASST training experience. Responses were rated as poor/neutral/satisfactory/good.

For physiotherapists, three questions were asked including; (1) In your opinion, did VASST improve safety aspects of treadmill training? (2) Did VASST reduce manpower requirements for safe treadmill training? (3) Would you like to use VASST in future? Responses to these three questions were rated yes/no/neutral.

### Protocol deviations

Due to non-remediable technical faults in the Gaitrite walkway halfway during the study, temporal gait measures such as cadence were not recordable. Two subjects could not obtain reliable HSL and NHSL readings at week 4 and 8.

### Statistical analyses

Descriptive statistics were used to summarize the baseline characteristics of subjects. The Wilcoxon signed-rank test was applied to evaluate the change from baseline at week 2, 4, and 8 on all outcome variables. The change between week 4 and 8 was examined as well. A statistical test was deemed significant if the *P* < 0.05. All statistical analyses were performed using STATA 10 (Statacorp).

## Results

### Baseline data (week 0)

Subjects were recruited from consecutive outpatient clinic visits at the ambulatory rehabilitation facility. Out of 147 subjects screened, 18 met eligibility criteria, eight declined consent and 10 were recruited. Table 1 shows the baseline demographic and clinical characteristics of the 10 subjects. The mean age was 55.5 years (*SD* = 9.8 years, median 56 years). Eight subjects were male and the mean duration poststroke was 2.19 years (*SD* = 1.52 years, median 1.74 years). Eight subjects suffered from intracerebral hemorrhages while two had ischemic strokes. All had previously undergone treadmill training during rehabilitation.

**Table 1:** **Baseline characteristics of subjects (*n* = 10)**.

	**Age (years)**	**Gender**	**Race**	**Days post-stroke**	**Stroke type**	**AL**	**WA**	**FAC**	Gait speed(m/s)^*^	Distance (m)^**^
1	54	M	C	260	L, BG, H	R	Nil	5	0.38	97.40
2	60	M	S	1957	L, BG, H	R	Nil	4	0.74	201.40
3	62	M	C	399	R, T, H	L	WS	5	0.65	120.00
4	56	M	C	1107	L, BG, H	R	AFO	5	0.74	230.00
5	43	M	M	214	L, BG, H	R	Nil	5	0.49	135.60
6	71	F	C	1222	R, CR, I	L	Nil	4	0.77	178.00
7	65	M	C	1176	R, BG, H	L	Nil	5	1.18	317.00
8	54	F	C	679	R, BG, H	L	WS	4	0.16	46.20
9	52	M	C	407	R, CR, I	L	Nil	5	1.00	286.50
10	38	M	I	589	L, BG, H	R	Nil	5	0.80	170.50

*Legend: M, Male; F, Female; I, Indian; C, Chinese; S, Sikh; M, Myanmarese; R, Right; L, Left; H, Hemorrhage; BG, Basal ganglia; T, Thalamic; CR, Corona radiata; I, Infarction; WS, Walking stick; AFO, Ankle foot orthosis; FAC, Functional ambulation category; WA, Walking aids; AL, Affected leg*.

* *Detemined using 10 m walk test measurements*.

** *Detemined using 6 min walk test measurements*.

All 10 subjects were community-dwelling with FAC ranging from 4 to 5. Walking aids were used in two subjects and ankle foot orthosis in one subject. At baseline, the mean gait speed by 10 mWT was 0.69 m/s (*SD* = 0.29 m/s) and mean distance walked by 6 mWT was 178.3 m (*SD* = 84.0 m) and BBS was 48.3 (*SD* = 3.13). Baseline mean hemiparetic step length (HSL) was 37.9 cm (*SD* = 13.2) NHSL was 37.2 cm (*SD* = 12.3 cm).

### Post VASST training results (week 4)

Post-training gains in 10 mWT (0.06 m/s) and 6 mWT (31.9 m) were seen at week 2 (*P* < 0.014, *P* < 0.007, respectively). Table 2 shows the gains across time after training.

**Table 2:** **Within group differences relative to baseline across time (*****N*** **= 10)**.

**Variable**	**Mean**	***SD***	**Median**	**IQR**	*P*-value^*^
**(1) WALKING SPEED (m/s)**					
Week 2–week 0	0.05	0.06	0.03	0.04	0.014
Week 4–week 0	0.06	0.08	0.06	0.07	0.037
Week 8–week 0	0.12	0.1	0.13	0.12	0.013
Week 8–week 4	0.06	0.07	0.06	0.13	0.041
**(2) WALKING CAPACITY(m)**					
Week 2–week 0	31.93	26.03	23.85	46.3	0.007
Week 4–week 0	54.26	30.91	48.6	51.6	0.005
Week 8–week 0	55.94	31.78	45.5	45.4	0.005
Week 8–week 4	1.68	21.58	0.5	22.9	0.838
**(3) BERG BALANCE SCALE (/56)**					
Week 2–week 0	1.5	1.84	1	3	0.042
Week 4–week 0	2	1.83	1	2	0.005
Week 8–week 0	2.3	2.21	2	3	0.01
Week 8–week 4	0.3	1.06	0	1	0.246
**(4) FUNCTIONAL AMBULATION CATEGORY**					
Week 2–week 0	0.1	0.32	0	0	0.317
Week 4–week 0	0.1	0.32	0	0	0.317
Week 8–week 0	0.1	0.32	0	0	0.317
Week 8–week 4	0	0	0	0	NA
**(5) HEMIPARETIC STEPLENGTH (mm)**					
Week 2–week 0	194.3	402.2	4.6	10.4	0.013
Week 4–week 0	104.1	303.4	9.01	11.04	0.005
Week 8–week 0	−90.3	541.8	3.01	6.88	0.333
**(6) NON-HEMIPARETIC STEPLENGTH (mm)**					
Week 2–week 0	196	401.7	6.65	8.6	0.005
Week 4–week 0	104.6	306	5.03	14.2	0.005
Week 8–week 0	−91.4	543	−0.13	5.61	0.959

* *P-values are obtained from Wilcoxon sign rank test*.

After 4 weeks of training upon completion of VASST training, 9/10 gained in walking distance (+54.3 m, *SD* = 30.9 m, *P* = 0.005) while 7/10 subjects had improvements in self-selected gait speed on their 10-m walk test (+0.06 m/s *SD* = 0.08 m/s, *P* = 0.037). There were also significant gains in BBS score of 2.0 (*SD* = 0.29, *P* = 0.007) (Table 2) At primary end point (week 4), both HSL and NHSL showed significant and similar mean gains of 104 mm compared to week 0. Compliance with all 12 training sessions was 100% for all subjects and there were no drop-outs or serious adverse events when VASST was used. Two subjects had self-limiting foot pain, which did not interrupt training. There were no falls during or following VASST training.

Ninety percent of subjects rated their VASST experience positively in terms of beneficial effects on walking independence, ability to walk longer distances and improved balance and 80% desired VASST training as part of their rehabilitation programme. Many subjects also reported that they were able to train at higher speeds compared to standard treadmill practice. The treating physiotherapist was of the opinion that VASST training increased safety compared to standard treadmill training and that it was a useful tool. However, manpower demands were not reduced.

### Post VASST training results (week 8)

At week 8, further gains in mean gait speed (+0.12 m/s *SD* = 0.1, *P* < 0.01) and mean walking capacity (+56.0 m, *SD* = 31.8, *P* = 0.005) were recorded a month after cessation of training (Table 2).

For gains in walking capacity (6 min WT), 1 subject had a reduction in 6 Min WT at week 8 and the rest either maintained or continued to increase in distance walked. For gait speed (10 mWT), only 1/5 slowed down at week 8 while the rest either maintained or increased self-selected gait speed at week 8 after training was stopped.

## Discussion

This feasibility study focused on safety, acceptability and preliminary clinical efficacy. Hence, subjects were initially selected for their independent walking abilities rather than initial speed. This was to ensure that a wide range of baseline walking speeds was included for VASST training. Overall analysis showed that this was a positive pilot study with high safety and acceptability from both trained subjects and their trainers. Seven of the 10 subjects demonstrated small but significant gains in walking speed (8.7%) and 90% of subjects had appreciable and significant gains in walking capacity (30%) which sustained gains at week 8 after cessation of training. There were striking differences between mean and median values in both HSL and NHSL values and this could be explained by the small sample size and presence of outliers. Both HSL and NHSL showed similar gains at week 4 after VASST interventions were completed, and this could be explained by entrainment of training effects on both affected and non-affected lower limbs present during stepping movements on VASST. However, gains for both HSL and NHSL were not sustained at the week 8, likely due to the short training duration of the study (12 sessions) and possibly a lack of follow on overground training after VASST.

In general, subjects who walked at <0.2 m/s failed to make appreciable gains in either walking speed or distance throughout the 8-week study period. While the majority gained in distance walked, and there was some variability as some faster ambulators (>1.1 m/s) did not improve in gait speed after VASST training. Gains in walking independence (FAC) and balance (BBS) were minimal due to ceiling effects as the majority were already community ambulators.

In general, treadmill training produces benefits in walking speed and distance, which last beyond the training period. When treadmill training was compared with no intervention/non-walking interventions, it produced 0.14 m/s faster walking speed and 40 m greater distance walked immediately after intervention and these benefits were maintained beyond the intervention period. Such training effects may result in clinically meaningful improvements such as a 1 point gain in the modified Rankin scale with a gain in walking speed of 0.16 m/s.

Our study drew similarities to Polese's meta analyses in terms of a similar duration of treadmill training (30 min) and frequency (3–4 times per week) for both subacute and chronic stroke subjects in either hospital or ambulatory setting (Polese et al., 2013). However, it was noted that there was some variability in the total duration of interventions with a mean of 9 weeks, *SD* = 7, thus our study was comparatively shorter in terms of duration of training. While VASST training produced better gains for walking distance, likely due to the increased number of steps entrained by the foot sensor feedback, it was inferior with regards to gains in gait speed. This could possibly be explained by a relatively shorter period of training and lack of non-walking interventions such as limb strengthening which may have positive effects on walking. We note however, sustained gains in walking capacity and speed at 8 weeks follow up and these were likely due to subjects taking a larger number of steps rather than bigger steps.

In comparison with Ada's AMBULATE trial, where treadmill training was commenced on 102 chronic stroke community ambulant patients (up to 5 years post stroke) in a 3 arm study of subjects undergoing 30 min of treadmill training thrice per week over 4 months or 2 months or controls with no intervention, mean gains in gait speed for patients undergoing 2 months duration of treadmill training was about ~22% (+0.11 m/s at 2 months) vs. 8.7% for our study; and mean improvement in 6 min walk test was about ~15% (+34 m) vs. 30.3% in VASST. In terms of step length, the AMBULATE trial saw patients undergoing 2 month treadmill training benefiting from a mean step length gain of ~20% after 4 months (Ada et al., 2013). This was similar to gains obtained in our study (24%).

Although their gains did not persist at 12 months follow up, it is likely that their longer training duration of 2 months and increasing amounts of over ground and stairs training accounted for the higher gains in walking speed compared to our 1 month VASST training protocol. It is possible that VASST training using specific foot placement feedback could also have resulted in many more steps undertaken during training, resulting in superior gains in walking distance despite relatively short programme duration, together with a speed-dependent training automated-algorithm which contributed to significant gains in step lengths and distance. It is also possible that VASST training using specific foot placement feedback counter could also have resulted in motivating subjects to catch up with the speed challenge imposed resulting in superior gains in walking distance despite a relatively shorter programme duration. In addition, the speed-dependent computer automated training algorithm was seen to generate a consistent and safe delivery of incremental speed challenges to our subjects, possibly contributing to improved training efficiency and efficacy.

Traditional treadmill training had previously relied on slow and steady speeds, however, faster treadmill training improves walking speed to a greater extent than self-selected speed post stroke without compromises to the gait pattern or increase in gait deviations (Tyrell et al., 2011). *Two* recent clinical trials examined “faster than self selected speeds” of treadmill walking and observed greater improvements in over ground walking speeds when participants were trained with faster than self-selected speeds. In addition, both stride length and cadence were increased to a greater extent following fast treadmill training post stroke (Pohl et al., 2002; Sullivan et al., 2002; Lau and Mak, 2011). Such a speed dependent algorithm was employed in VASST treadmill system by encouraging subjects to continuously trigger the proximal foot sensors most of the time, before treadmill speed was automatically increased.

Several study limitations are highlighted. Firstly, the small sample size and lack of a control group (non-treadmill training/non-walking intervention) opens the possibility of selection bias and non-treatment effects. The baseline demographics of our sample were dissimilar to the local stroke population as they had a median age of 55 years, male: female ratio of 4:1 and 80% of the cohort were diagnosed with hemorrhagic strokes. This sample was atypical when compared with the local stroke population (Eich et al., 2004), where the median age was 68 years, male: female ratio was 1.3:1 and hemorrhagic strokes made up 20% of the total stroke population. Thus, our results would lack generalizability to the general stroke population.

The relatively short length of follow up does not allow conclusions to be made with regards to long-term effects related to VASST training. Ada et al. noted that while 4 month programmes were superior to 2 month programmes in terms of distance walked and walking speed vs. no interventions, these differential effects related to training duration were lost in both intervention groups at 1 year follow up (Ada et al., 2013). This highlights the importance of continuing exercise programmes after treadmill training. Eich et al. (2004) had suggested that addition of task specific training such as walking practice to treadmill training in moderate stroke-severity patients was superior to similar duration of standard physical therapy. Such a combination was not undertaken for this initial study, as we wanted to ascertain the clinical effects of VASST in isolation. Nevertheless, the robust maintenance of short-term gains in walking speed and gait after 2 months could be explained by training effects on muscle and aerobic capacity and subjects having increased walking practice in the community possibly due to better motivation or confidence to ambulate.

The authors are of the opinion that VASST training may be superior with regard to patient autonomy and feedback in terms of foot position on the belt, safe graded progression of training speed and fall risk reduction by ameliorating the possibility of backward displacement in addition to a safety harness. Thus, the work of the physiotherapist in ensuring safety during treadmill training is facilitated thus allowing greater attention to gait pattern and safe and consistent exercise progression. The high level of user acceptance positions VASST as a potential innovative alternative to the current established methods used in treadmill training.

## Conclusion

In conclusion, results from this preliminary study show that the usage of a speed sensing treadmill system for gait training of ambulant chronic stroke patients is feasible, effective and safe. Outcomes following VASST training compare favorably with standard treadmill protocols, particularly in terms of improvements in walking distance followed by walking speed with regard to slow community ambulators >0.2 m/s. A larger randomized study to compare VASST with standard treadmill training in combination with standard gait training protocols is thus warranted.

## Conflict of interest statement

The authors declare that the research was conducted in the absence of any commercial or financial relationships that could be construed as a potential conflict of interest.
